# Influence of motivational placebo-related factors on the effects of exercise treatment in depressive adolescents

**DOI:** 10.1007/s00787-021-01742-5

**Published:** 2021-03-12

**Authors:** Heidrun Lioba Wunram, Stefanie Hamacher, Max Oberste, Susanne Neufang, Luisa Belke, Franziska Jänicke, Christine Graf, Eckhard Schönau, Stephan Bender, Oliver Fricke

**Affiliations:** 1 Department of Child and Adolescent Psychiatry Psychosomatic and Psychotherapy, University Hospital of Cologne, Cologne, Germany; 2 Department of Medical Statistics, Informatics and Epidemiology (IMSIE), University of Cologne, Cologne, Germany; 3 Department for Molecular and Cellular Sports Medicine, German Sport University Cologne, Cologne, Germany; 4 Department of Psychiatry and Psychotherapy, Medical Faculty Heinrich-Heine University, Düsseldorf, Germany; 5 Children’s Hospital Amsterdam Street, Cologne, Germany; 6 Clinic for Psychiatry Marienborn, Düren, Germany; 7 Institute of Movement and Neuroscience, German Sport University Cologne, Cologne, Germany; 8 Children’s Hospital, University Hospital of Cologne and UniReha®, University Hospital of Cologne, Cologne, Germany; 9 Department of Child and Adolescent Psychiatry, Psychotherapy and Child Neurology, Gemeinschaftskrankenhaus Herdecke and Chairs of Child and Adolescent Psychiatry, Witten/Herdecke University, Witten, Germany

**Keywords:** Physical activity, Exercise, Adolescent depression, Motivation, Enjoyment, Placebo

## Abstract

Recent meta-analyses reveal a moderate effect of physical activity (PA) in the treatment of adolescent depression. However, not only the underlying neurobiological mechanisms, also the influences of placebo-related motivational factors (beliefs and expectancies in sporting, enjoyment and prior sports experiences), are still unclear. Based on the data of our prior study “Mood Vibes”, we hypothesized that placebo-inherent factors like positive prior sports experiences and motivational factors, (positive beliefs, expectancies, and enjoyment related to PA), would increase the effects of an add-on exercise-therapy in juvenile depression. From 64 included depressed adolescents, 41 underwent an intensive add-on PA-therapy. Motivational factors were assessed using sport-specific scales. The changes in depression scores under treatment were rated by self-rating scale (German “Childhood Depression Inventory”, (DIKJ)). A mixed model for repeated measures (MMRM) was used to analyze the effects of the different motivational variates on DIKJ. While prior sports experiences had no impact, motivational factors showed a significant effect on PA-induced changes in DIKJ scores (*p* = 0.002). The demotivated participants improved less, whereas it was sufficient to be neutral towards sporting to benefit significantly more. Motivational placebo-related factors (beliefs, expectancies and enjoyment regarding PA) affected the outcomes of an exercise treatment in depressed adolescents. Yet, a neutral mindset was sufficient to profit more from PA. Prior sporting in the sense of positive conditioning and as a protective factor did not play a role. Knowledge about these influences could in a second step help to develop tailored therapies.

**Supplementary Information:**

The online version contains supplementary material available at 10.1007/s00787-021-01742-5.

## Introduction

Adolescent depression is a growing part of the burden of disease worldwide [1]. Prevalence rates differ depending on the countries included and the type of studies [2, 3]. Even if some deny an increased prevalence over the past 30 years, they agree that there is growing awareness of adolescent depression, which presumably was long time under-diagnosed by clinicians [3]. Taking into account the impact of the disease in the personal and professional development of adolescents, effective treatment is particularly important [4]. Chronicity in adulthood and an elevated risk of suicide are major risk factors [5]. As adolescence is a period of constant biological changes in the purpose of development, mechanisms of pathology and treatment differ from those in adults [6]. In most countries, guideline recommended treatment options for adolescent depression are cognitive behavioral therapy (CBT) and pharmacotherapy, or the combination of both [7–9]. The greatest evidence in antidepressant drug treatment is established for selective serotonin reuptake inhibitors (SSRI), but there are far less options in approved medicaments than in adulthood [10].

Physical activity (PA) for the treatment of adult depression has gained growing interest in research, clinical treatment and guidelines [7, 8]. A Cochrane review from 2013 found a moderate effect of physical exercise in comparison to no treatment or a control condition. Additionally when compared to psychotherapy and pharmacological interventions, physical exercise was equally effective [11]. Regarding physical exercise as treatment option for depressed adolescents, the state of research is still scarce [12]. Oberste and colleagues found in a recent meta-analysis, including nine studies, a moderate anti-depressant effect of PA compared to control treatments, with a confidence interval that ranges from a small up to a large effect [12]. In the limitations section, the authors state that low methodological quality and potential placebo related factors in included studies might have influenced the effect.

Motivational placebo-related factors (beliefs, expectations, expectancies, prior experiences etc.) are known to have an impact on psychotherapeutic and pharmacological treatment in depression [13]. Regarding motivational aspects of exercise treatment in depression, literature is less abundant [14]. Moreover, there are far less studies focusing on placebo factors in children and adolescents [15]. Even though age and developmental aspects are specifically interesting in this matter [16, 17].

### Placebo related influences in psychotherapeutic and pharmacological treatment of depression

Since the publication of Beecher’s article “The powerful placebo” in the Journal of the American Medical Association (JAMA) in 1955, randomized clinical trials (RCTs) are the scientific gold-standard to exclude placebo effects as much as possible in the evaluation of interventions [18]. However, even applying the methodological techniques of a RCT, some psychological confounders and placebo effects cannot be excluded. Major psychological mechanisms of the placebo-related responses are beliefs and expectations, expectancies (as a subset of the prior) and classical conditioning [19, 20]. Expectation is a conscious cognitive process including a positive anticipation of the effect of an applied treatment [21–23]. Whereas negative anticipation and cognition concerning the effects of a treatment generate the so called “nocebo effect” [21]. Expectancies in the sense of more subconscious psychophysical anticipations, are conceptualized by some placebo and nocebo researchers as a subset of expectations [24–26]. In line with this distinction, we focused in the present study on expectations in the sense of a verbalized and measurable construct [26].

One possibility to increase positive expectation, is adding positive verbal information to a treatment. The activation of neuronal activity in verbal memory processing brain areas seem to lead to an activation of emotion processing brain areas [13]. The other psychological mechanism associated with a placebo-inherent effect is classical conditioning. For instance, we can see higher positive (placebo-related) response if a drug is administered after previously effective experiences with an actual analgesic drug [27].

Studies have analyzed placebo-related response from the physiological point of view, for instance, in different drug treatments of depression. Antidepressant treatment activated the release of endogenous opioids, dopamine and cannabinoids, and serotonergic pathways [13]. Oxytocin and nitric oxide (NO) were also found to influence placebo response [28]. Neuroimaging techniques can show possible brain areas activated under placebo treatment [29]. The influence of motivation and expectations on dopaminergic pathways was depicted in a neuroimaging study, where dopamine was released in the nucleus accumbens [13]. A more profound understanding of the neurobiological and neurophysiological mechanisms of expectation and conditioning of placebo effects could help to harness this knowledge for treatment interventions in depression [30]. There is vast research in adults on expectations and conditioning associated with placebo effects in the different medical conditions and treatments [19]. Age and developmental specificities seem to influence the placebo and nocebo effects in children and adolescents [15, 16, 31]. Younger age was found to increase susceptibility to placebo responses [17]. Higher capacities of associative learning, stronger suggestibility and placebo by proxy are some of the discussed particularities [15, 32].

### Motivational placebo-related influences in exercise treatment of depression

There are some studies dealing with motivation, expectancies, beliefs and conditioning (inherent to placebo effect) in exercise and physical performance [33, 34] as well as with psychological benefits of exercise [14]. Placebos are believed to have a small to moderate effect on exercise performances [14, 34]. Some authors even argue that psychological or therapeutic benefits of acute exercise are mostly due to placebo because of the fact, that there is no evidence for a “standard” duration or intensity needed, to have the desired medical or psychological effect [35]. Double-blind studies in exercise interventions are impossible to conduct, and most studies fail to include a three-armed design with an intervention group, an active placebo-intervention group and an inactive control to filter out the placebo factors [14]. Taking into account that placebo is defined as a combination of expectation and conditioning, the motivation of the patient to reach a certain psychological amelioration by exercise is crucial. Beside the motivational expectancies, prior positive sports experiences can influence the conditioning effect. Mothes et al. examined the influences of habitual expectancies and induced expectancies on the psychological outcomes of exercise [36]. The participants with higher positive habitual expectations showed more psychological benefits in mood, enjoyment and decreasing anxiety [36]. Mothe and colleagues did not find the same effect for induced expectation, which though was detected by Helfer and colleagues who found an amelioration of post-exercise mood due to a manipulation of the expectancies [37].

### Placebo-inherent effects in the study “Mood Vibes”: the influences of belief/expectancies and prior sports experiences

The interventional study “Mood Vibes” analyzed two different types of PA as add-on therapies, compared to treatment as usual (TAU) in an inpatient treatment of depressed adolescents [38]. The main objective of the study was to establish evidence for an exercise intervention which requires less active efforts. It was conducted at the Department of Child and Adolescent Psychiatry of the University Hospital of Cologne over 2 years. One intervention was an active endurance training on a cycling ergometer and the other was a rather passive activity, on a whole body vibration device (WBV). The WBV-intervention is easy to perform for adolescents with a low urge to exercise. Therefore, it was expected to be an easily accessible alternative PA in depressive and rather passive adolescents. Depressed adolescents mostly have a low urge to participate in active sports disciplines [39]. It was hypothesized that WBV would be as effective as endurance cycling in its clinical effects, measured by a decrease in depression scores in the “Depressions Inventar für Kinder und Jugendliche” (DIKJ) at weeks 6, 14 and 26 after inclusion. Both exercise interventions were expected to be superior to TAU only.

The depressed adolescent inpatients were supposed to have a lack of motivation to exercise and, therefore, a lower positive outcome expectation with regard to an antidepressant effect of PA, compared to non-depressed. The potential antidepressant effect of the exercise-treatment was explained by the study staff before the intervention. It was hypothesized, that a lack of positive beliefs and expectancies regarding the effect of the exercise intervention would result in a lower decrease in depression scores. Secondly, it was expected that depressive adolescents would have a low grade of prior experiences in exercising and thus a minor positive conditioning regarding the exercise interventions. As secondary variables, the influences of enjoyment and perceived competence, were analyzed, though not as part of the placebo-related factors. Moreover, the influences of the placebo-like factors on physiological outcomes (spiroergometry, mechanography, serological parameters) were examined.

The objective of analyzing the psychological influences/ placebo-adherent factors, was to use this understanding for tailored treatment and if possible, to maximize treatment effects with this knowledge in the future [38].

## Procedures and measures

Detailed information about study design, participants, training methods, procedures and measures can be found in Wunram et al., 2018 [38]. In the present article, only relevant key-points for the topic will be mentioned.

### Study design and participant recruitment

#### Study design

In the longitudinal study, intervention groups had to perform PA during 6 weeks adjuvant to treatment-as-usual (TAU), 3–5 days a week for 30 min, respectively. The control group received TAU only. Physiological and psychological measurements were taken before intervention, after 6 weeks exercising and again after weeks 14 and 26 (supplement, tableA1). The motivations, expectancies and prior sports experiences (“Motivations and barriers to sports—MBS”) were assessed at baseline. The enjoyment scales (MSES) were completed by the two intervention groups, after completing the intervention at week 6. The control group participated in the same measurements as the intervention groups, except the MSES and the Feedback Questionnaire (FBQ). The study protocol was approved for the intervention by the University of Cologne Ethics Committee and has been officially registered in the German Clinical Trials Register in Freiburg under the identification DRKS00005120. Extern monitoring according to protocol and regulatory requirements was executed as initiation and close-out visit.

#### Participant recruitment

Participants were recruited from the inpatient units at the Department of Child and Adolescent Psychiatry of the University Hospital of Cologne, from July 2013 to July 2015. They were naïve for long-term medication. At admission to the inpatient treatment, adolescents and parents were screened for eligibility. The participants were included consecutively.

#### Inclusion criteria

Participants had to be between 13 and 18 years and meet DSM-IV/-5 and ICD-10 criteria of non-psychotic major depressive disorder (MDD), assessed by clinician rating with the Structured Clinical Interview for DSM-IV Axis I Disorders, German version (SKID-I) [40–42]. Additionally, patients needed a baseline score in the “Depressionsinventar für Kinder und Jugendliche” (“Depression Inventory for Children and Adolescents”, DIKJ) [43] of at least 18. They had to be of normal intelligence (i.e., IQ > 70 based on prior testing by Kaufman Assessment Battery for Children (K-ABC) [44] or Wechsler Intelligence Scale for Children (WISC)/ Hamburg-Wechsler-Intelligenztest für Kinder (HAWIK) [45]) and German language and reading were required. Sport aptitude and a spiroergometry with electrocardiography (ECG) was carried out before starting the intervention, following the official guidelines of the Society of Pediatric Sports Medicine [46]. Both genders were included. Comorbidities were allowed as long as not being an exclusion criteria.

#### Exclusion criteria

Adolescents were excluded if they suffered from one of the following conditions: schizophrenia, other psychotic disorders or psychosis in the medical history, including bipolar I and II disorder, severe borderline personality disorder, pervasive developmental disorder or current substance abuse, malignant diseases, a Body Mass Index (BMI) < 16 kg/m^2^, diseases causing restrictions to PA and the use of WBV. Permanent long-term psychiatric medication or medication with inherent psychotropic effects was not allowed.

#### Randomization

After written consent (adolescents/ parents/ legal guardians) to participate in the study, the Structured Clinical Interview for DSM-IV Axis I Disorders, German version (SKID-I) was performed to confirm diagnosis, check comorbidities and exclusion criteria, and evaluate current severity of the depressive disorder [42]. Randomization of vibration device and bicycle ergometer (1:1 ratio, permuted blocks of varying length) was implemented based on sealed, opaque envelopes by the Institute of Medical Statistics, Informatics and Epidemiology of the University of Cologne (IMSIE). The randomization sequence was generated using SPSS Statistics software (random number seed). The adolescents that were willing to participate in the measurements but not in the PA intervention were recruited for the control (TAU) group.

### Procedures and measures

Primary outcome (clinical effects on depression severity) and secondary outcomes (psychological placebo-like factors as motivation, expectancies, prior sports experiences, enjoyment and perceived competence), were assessed with the questionnaires described below.

#### Depression inventory for children and adolescents (DIKJ)

The German “Depression Inventory for Children and Adolescents” (DIKJ) was used as primary outcome measure for depression. It is a self-report questionnaire conceived on the basis of the Anglo-Saxon children’s depression inventory (CDI) [47]. The DIKJ assesses all fundamental symptoms of depression according to the criteria of major depression in the DSM-IV. Using unselected student samples Stiensmeier, Schürmann and Duda (1989) found an internal consistency between 0.85 > α ≥ 0.82 with a tendency for higher statistical values in older age samples [43]. In clinically conspicuous children and adolescents, the internal consistency lies at α ≥ 0.91 [43]. A raw-score of 18, indicator of a moderate depression severity, was set as cut-off criteria for inclusion into the study.

#### Beck depression inventory (BDI-II)

The Beck Depression Inventory (BDI-II) is a multiple-choice self-report inventory, with an age range from 13 years and over. It consists of 21 items that assess the severity of depression. Each item is a list of four statements arranged in increasing severity in alignment with DSM-IV criteria, relating to symptoms of depression (e.g., hopelessness, irritability, guilt, fatigue, weight loss etc.) [48]. The test has a good 1-week test–retest reliability (Pearson *r* = 0.93), and an internal consistency of α = 0.91. A raw-score between 20 and 28 points was taken as indicator of a moderate depression as it was suggested by Steer et al. [48].

#### Structured clinical interview for DSM-IV

Clinical rating of depression and assessment of comorbid disorders was performed using the German version of the Structured Clinical Interview for DSM-IV “Strukturiertes Klinisches Interview für DSM-IV” (SKID-I). The SKID-I serves to determine and diagnose psychic syndromes and disorders based on definitions of Axis I disorders within the DSM-IV (2000) 4^th^ ed., it is a structured interview lasting approximately 60 min [42].

#### Questionnaire “motivational factors and barriers to sports” (MBS)

All participants completed the following form regarding the placebo-like factors as motivation, beliefs, expectation and prior sports experiences: “Motivational Factors and Barriers to Sports - MBS”. It assesses quantity and quality of the adolescent’s physical activity before the inclusion in the study Mood Vibes. It covers the beliefs and expectations regarding the effects of different types of exercise as well as obstacles or reasons for not performing PA. The answers are rated on a Likert Scale that ranges from 1 (“completely correct”) to 5 (“not correct at all”). The extent of time needed for completion was approximately 15 min. The age range is between 13 and 18 years. The questionnaire was developed by Graf et al. out of the factors found by Sallis et al. and the CHILT-FB questionnaire [49–52]. The MBS is still in the process of validation in clinical and non-clinical samples (part of two doctoral thesis, results are not achieved yet).

#### Magglinger sports enjoyment scale (MSES)

The MSES was constructed on the basis of the English PACES questionnaire (Physical Activity Enjoyment Scale) from Kendzierski et al. and the German “Befindlichkeitsskalen” (BFS; State Scale) from Abele and Brehm [53–55]. It includes concepts developed by Scanlan et al. and McCarthy et al. about sport-commitment and sport-enjoyment as important factors of how to motivate people to do exercise [56, 57]. It comprises 20 items on five scales (perceived competence, social interaction, specific experience of movements, enjoyment of activity, and positive interaction with trainer). The items are rated on a 7-point Likert scale (ranging from 1 = never/not at all to 7 = completely right/very much). In non-clinical samples, its internal consistency is stated between C*ronbach’s alpha* 0.95 > α ≥ 0.75. It was filled in after the 6-week intervention.

#### Feedback questionnaire (FBQ)

The self-designed Feedback (FBQ) questionnaire from the study’s working group is comprised of 12 closed-ended questions rating the acceptance and criticism of the exercise interventions and the subjective motivations of the participants. Rating was possible on a 4-point scale ranging from 0 = never/not at all to 3 = completely right/always. Additionally, subjects could fill in three open questions with regard to the program.

#### Physical examination and measurements

Participants included into the study performed the following physical examinations before starting the intervention (t0), after 6-week intervention (t1) and after 8 weeks without exercise-intervention (t2):

- Spiroergometry (using systems *ZAN*^*®*^*, Blue Cherry and ergometer-cycle ergoline*^*®*^) with electrocardiography (*AMEDTEC ECG pro*^*®*^), including measurement of lactate and blood gas analysis, VO2 (maximal aerobic capacity), VE (respiratory minute volume), RER (respiratory quotient), HR (heart rate), RR (arterial blood pressure), assessing the maximal wattage per kilogram;
- Calipermetry (standardized estimation of body fat by skinfold caliper Siber-Hegner® measuring Scapula and Triceps skinfolds of left body side;
- Anthropometric measurements (height, weight, Body Mass Index (BMI), determined using clinical standard stadiometer and digital electronic scales;
- Mechanography assessing peak jump force (PJF), and peak jump power (PJP), with the Leonardo^®^ Jumping Platform (Novotec GmbH, Pforzheim, Germany).

Spiroergometry was done according to the german spiroergometry guidelines [58]. Mechanography testing was done according to Novotec^®^ handbook instructions [59, 60].

## Intervention

### Training procedures

The training of the two intervention groups took place daily from five to six p.m. in the training facilities of the rehabilitation and physiotherapy training rooms of the University Hospital of Cologne (Unireha^®^). Two members of the study staff supervised the training and controlled for correct execution of the physical activities. The size of the training groups differed as the participants were included consecutively. Participants could choose one training-free day weekly and exceptionally two training-free days, if in the whole 6 weeks the number of trainings equaled 18 sessions. The maximum achievable number of trainings was 30. If not at least 18 sessions in 6 weeks were completed, the participant was counted as intention to treat (ITT).

### Vibration plate (WBV)

The WBV was executed on the Galileo^®^ training device (model Advanced Plus) from Novotec Medical GmbH. The vibration plate stimulates a movement pattern similar to human gait. The training principle is based on the activation of proprioceptive spinal circuits, inducing a certain number of stretch reflex contractions per second depending of the frequency chosen. The training improves muscle power and coordination of the legs, the hip and partly of the trunk. It has only a small effect on the cardiovascular system. It is used in rehabilitation and training in different areas, one pilot study covering therapy of stereotypies in autism [61–63]. The side-alternating vibrations generated by Galileo^®^ can be continuously varied in amplitude and frequency and the training load is determined by those parameters. The adolescents of the WBV group were trained at a frequency of 20 Hz and an amplitude of 2 mm. The first 12 days, the length of the six exercises on the plate was of two minutes, then three minutes. Between the stimulations was an equal pausing time, necessary for the recovery of the muscle strength. This means that by WBV technique muscle contractions as walking a distance of 15.840 steps in the first 12 days, and 23.760 steps in the following sessions, was executed. Additionally, the six standardized exercises on the plate comprised contractions of the arms and shoulder, rotation of the trunk, varieties of leg positions and squats (supplement, tableA4).

### Ergometer training

The ergometer training took place in the premises of Unireha^®^ GmbH on stationary cycles from the firm Ergosana^®^ and was supervised by the study personnel. In cooperation with the German Sport University Cologne a 30-min interval training calculated on the maximal performance in the previous spiroergometry results was applied (supplement, tableA6). Completion of each participant’s protocol was supervised by the training staff.

### Treatment as usual (TAU)

Participants in the TAU condition followed their therapy schedule at the inpatient units of the Department of Child and Adolescent Psychiatry, Psychosomatic and Psychotherapy, Cologne. Common therapy offers were psychotherapy in form of individual sessions with a psychotherapist or psychiatrist, group psychotherapy sessions, exercise therapy, art therapy and music therapy. The intervention groups received also TAU, the exercise therapy was adjuvant.

### Statistical analysis

Analyses was carried out according to the modified intention-to-treat (mITT) approach, i.e., including all randomised patients with at least two valid assessments and having participated in at least one training session. Subjects that started medication in the course of the study and administered for longer than 3 weeks were also considered ITT. Drop Outs were classified as subjects that did not participate in t1 [second assessment of measurements). Patient data were summarized using count (percentage), mean ± standard deviation (SD) or median (interquartile range (IQR)], contingent on distributional characteristics. Normality of empirical distributions was formally evaluated by the Shapiro–Wilk test (at 10% significance level). Separate mixed models for repeated measures (MMRM) were used to analyse the effects of motivation, prior sports experiences (activity) and enjoyment (MSES) on DIKJ, respectively. DIKJ raw scores were the dependent variables, fixed effects were treatment, time, interaction treatment*time, sex, age and additionally motivation, activity or MSES, respectively (type III sums of squares, ARH1 covariance structure over time). The overall multilevel model was significant. Secondary endpoints as BDI-II and physiological parameters were analysed along the same line. Regarding the DIKJ and BDI-II scores calculations were effected over 26 weeks (baseline to week 26), regarding the physiological parameters, calculations were effected over 14 weeks (baseline to week 14, i.e., last measurement of spiroergometry and mechanography). Potentially confounding variables were evaluated in a stepwise manner (in- and exclusion from the model equation, e.g., covariates as medication, number of trainings, total therapy times, additional sports). Influence of motivation or treatment (without control) on MSES was analysed using univariate ANOVA. All reported p-values are two-sided and considered statistically significant if ≤ 5%. All calculations were performed using SPSS Statistics 26 (IBM Corp., Armonk, NY, USA).

## Results

### Subjects included

Sixty-four out of 89 screened patients met criteria for study eligibility and 41 participants were randomized to the intervention condition, whereas 23 were included in the TAU non-randomized control condition.

### Drop outs and intention to treat

As ITT were analyzed eight subjects (*n* = 6 from control, *n* = 1 from WBV and *n* = 1 Ergometer). Twelve subjectsdropped out prior to t1 (WBV = 3; ergometer = 3, control = 6) because of anticipated discharge (2 of each group) or retracted consent to participate (WBV = 1, ergometer = 1, control = 4). The total number of participants at t2 was 48 (ergometer = 16, WBV = 18, control = 14) and 39 at follow-up (post t2: ergometer = 14, WBV = 13, control = 12). Some questionnairescould not be included in the analysis as they were either not completed or not handed in.

### Demographic and clinical characteristics of the sample

Eighteen boys (28.1%) participated in the study (supplement, tableA2) Mean age was 15.88 ± 1.15 years, IQ assessed by WISC IV with 100.12 ± 11.94 in the average. BMI was with percentiles 68.3 ± 31.9 borderline between normal and overweight. The most frequent comorbidities in the sample were anxiety [*n* = 14 (21.9%)] and somatoform disorders [*n* = 8 (12.5%)], (supplement, tableA2). DIKJ mean scores at baseline did not differ significantly between intervention and control groups (27.6 ± 6.4 DIKJ score/entire sample) (supplement, tableA2). *P* values derived from one-way ANOVA for quantitative variables and Fisher’s exact test for qualitative variables. Patients were supposed to be medication naïve. Those who nevertheless received medication (pro re nata, PRN) with psychotropic effects in the course of the study for longer than 3 weeks were treated as ITT. Introduced in the mixed model analysis, medication showed no statistically significant influence. TAU-therapies were quantified for each patient of every therapy form offered in the ward (Psychotherapy, Group Therapy, Arts Therapy, Sports Therapy, Music Therapy, Social Work interventions). Total Psychotherapy in minutes weeks 1–6 was almost equal in all groups (mean = 311 ± 173). Mean length of stay was 68 ± 33 days for the entire sample. Adherence to sports program and additional sports therapy time did not differ significantly and introduced in the mixed model analysis did not show to have an effect on depression scores.

### Prior sports experience (MBS)

The adolescents were allocated to three different activity groups, following the history of PA in the past. Three activity groups were defined as follows: very active group A (more than four sessions of PA > 30 min/week), regularly active group B (2 to 4 sessions of PA > 30 min/week) and less active group C (less than two sessions of PA > 30 min/week). All types of PA mentioned in the questionnaire were taken into account, school sports were not included. The depression scores of the three activity groups were compared with *Kruskal–Wallis-Test*, as in the *Kolmogorov–Smirnov-Test* no normal distribution was found (*p* = 0.015). The test-statistics were asymptotic *Chi-square* distributed. The allocation of the activity groups in the control, WBV and ergometer groups was not statistically significantly different (*p* = 0.279, Table 1). Also, the baseline DIKJ raw scores did not differ significantly in the activity groups (Table 1).

**Table 1:** Descriptive of Activity groups and Motivation groups

	Group A	Group B	Group C	*p* value	Group „motivation “	Group „neutral “	Group „demotivation“	*p* value
Total sample	*n* = 14 (29.2%)	*n* = 10 (20.8%)	*n* = 24 (50.0%)		*n* = 21 (39.6%)	*n* = 13 (24.5%)	*n* = 19 (35.8%)	
Intervention				0.279				0.255
Ergometer	*n* = 7 (43.8%)	*n* = 2 (12.5%)	*n* = 7 (43.8%)		*n* = 10 (55.6%)	*n* = 3 (16.7%)	*n* = 5 (27.8%)	
WBV	*n* = 2 (11.8%)	*n* = 4 (23.5%)	*n* = 11 (64.7%)		*n* = 7 (38.9%)	*n* = 6 (33.3%)	*n* = 5 (27.8%)	
Controls (TAU)	*n* = 5 (33.3%)	*n* = 4 (26.7%)	*n* = 6 (37.5%)		*n* = 4 (23.5%)	*n* = 4 (23.5%)	*n* = 9 (52.9%)	
DIKJ- raw score (t0)	28.5 ± 6.5	27.9 ± 3.2	27.3 ± 7.2	0.892	26.05 ± 10.2	29. 00 ± 12.23	33.11 ± 12.51	0.112
BDI II raw score (t0)	31.13 ± 11. 2	28.38 ± 11.7	29.71 ± 11.8	0.803	28.1 ± 10.2	29.00 ± 12.2	33.1 ± 12.5	0.291

Activity groups and Motivation groups with DIKJ- and BDI-scores at t0, mean and SD, calculated with Fisher’s exact test and Kruskal–Wallis test

Group A: very active (> 4 × 30 min/week); Group B: active (2–4 × 30 min/week); Group C: less active (< 2 × 30 min/week)

### Expectancies and other motivational factors (MBS)

The extent of positive beliefs and expectancies regarding the effects of exercise was clustered in three groups: the group “motivation” (relation of the scores motivation/ demotivation < 0.9), the group “neutral” (relation of the scores motivation/demotivation 0.9–1.1) and the group “demotivation” (relation of the scores motivation/demotivation > 1.1). The scores were calculated following the answers in the sections V. “Reasons in favor of physical activity” and VI. “Reasons against physical activity” of the questionnaire “Motivations and barriers to sports” [52]. In the last section of the questionnaire (VII. “What would motivate you to do more sports”), further motivational reasons to exercise were assessed. The distribution of the motivation, demotivation and neutral group in the ergometer, WBV and control group, did not differ significantly (*p* = 0.255, Table 2).

**Table 2:** MSES subscales and sum score

	Group	*N*	Mean	SD	Median	IQR	*p*
Perceived competence							
Ergometer		17	4.16	1.49	4.75	2.50	0.049
WBV		18	3.13	1.55	2.50	2.75	
Social Interaction							
Ergometer		17	4.34	1.56	4.75	2.63	0.227
WBV		18	3.69	1.58	4.00	2.94	
Specific Movement experience							
Ergometer		17	4.44	1.39	5.00	1.88	0.042
WBV		18	3.49	1.41	3.63	2.50	
Positive Interaction with the trainer							
Ergometer		17	4.48	1.47	4.50	1.75	0.345
WBV		18	4.06	1.40	4.13	2.81	
Enjoyment							
Ergometer		17	4.87	1.55	4.75	1.63	0.215
WBV		18	4.03	1.90	4.00	4.00	
MSES sum score							
Ergometer		17	22.34	6.48	22.75	8.13	0.102
WBV		18	18.35	7.00	18.75	11.88	

p from Mann–Whitney-U- test; IQR: Interquartile Range

### Scores of Magglinger Sports Enjoyment Scale (MSES) and Feedback Questionnaire (FBQ)

In the MSES sum score group differences of the Enjoyment Scale were not statistically different (*p* = 0.215, Table 2). For the Scales “Perceived Competence” and “Specific Movement Experience”, Ergometer was rated higher compared to WBV (*p* = 0.049 and *p* = 0.042). In the FBQ, groups did not differ significantly. There was a trend towards higher scores in the WBV group with respect to the item “Got motivated to continue exercise” (supplement, table A12).

### Influence of prior sports experiences on DIKJ and BDI-II over time

Mean DIKJ and BDI-II scores showed no significant differences at baseline in the activity groups (Table 1). Also in the longitudinal analysis over all measurements, activity groups had no influence on DIKJ or BDI-II decrease (Table 3).

**Table 3:** Fixed Effects of Activity Groups on DIKJ and BDI-II raw scores over time (Mixed Model Analysis)

Source	DIKJ raw score *p* value	BDI-II raw score *p* value
Intercept	0.069	0.420
Treatment group	0.462	0.485
Time	0.000	< 0.001
Age	0.866	0.811
Gender	0.152	0.022
Treatment group* time	0.338	0.285
Activity group	0.933	0.913
Time * activity group	0.809	0.887

Time * activity group = activity group over time

### Influence of Motivation on DIKJ and BDI-II over time

**Fig. 1:**
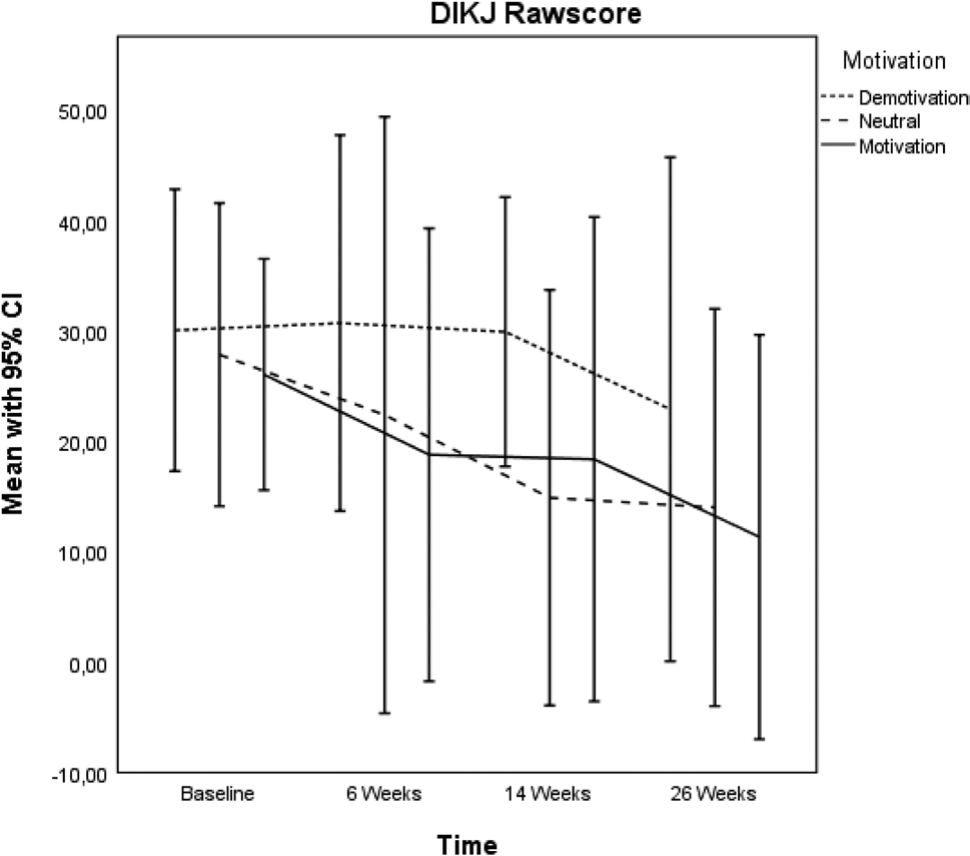
DIKJ-raw score pairwise comparisons/ time

Motivation groups showed no significant differences in DIKJ and BDI-II scores at baseline (Table 1). However, in the longitudinal analysis over all measurements motivation groups showed significant influence on DIKJ (*p* = 0.002) and BDI-II scores (*p* = 0.040), demonstrated in the interaction time*motivation group (Table 4, Fig. 1). In the pairwise comparisons, the DIKJ scores in the Neutral group were on average 8.95 points lower, and in the Motivation group 8.17 points lower, compared to the Demotivation group (Table 5). Concerning BDI-II, results missed closely significance in the pairwise comparisons (*p* = 0.065). The raw scores in the Demotivation group were 6.64 points higher compared to the Neutral group, and 6.74 points compared to the Motivation group (Table 5).

**Table 4:** Fixed effects of motivation group on DIKJ and BDI-II (Mixed Model Analysis)

Source	DIKJ raw score *p* value	BDI-II raw score *p* value
Intercept	0.370	0.994
Treatment group	0.303	0.286
Time	< .001	< 0.001
Age	0.322	0.261
Gender	0.148	0.008
Treatment group* time	0.475	0.579
Motivation group	0.002	0.121
Time * motivation group	0.002	0.040

Time * motivation group = motivation group over time

**Table 5:** Pairwise comparisons of Motivation groups over time for DIKJ and BDI II (Mixed Model Analysis)

	Pairwise comparisons					
	(I) motivation	(J) motivation	Mean difference (I–J)	95% confidence interval for difference^c^		*p* value
DIKJ	Demotivation	Neutral	8.948^*^	3.488	14.408	0.002
		Motivation	8.166^*^	3.059	13.274	0.002
BDI II	Demotivation	Neutral	6.638	− 0.937	14.212	0.084
		Motivation	6.741	− 0.444	13.926	0.065

Based on estimated marginal means. *The mean difference is significant at the 0.05 level

### Influence of Motivation on physiological parameters over time

Motivation groups showed significant influences on spiroergometry performance outcomes over time (14 weeks, last measurement of spiroergometry and mechanography). Maximal Wattage/kg (Watt/kg) and RER peak showed significant effects, *p* = 0.029 and *p* = 0.005, respectively (Table 6). Demotivated and neutral groups showed significant less performance ameliorations compared to the motivated group (Table A7, Table 7).

**Table 6:** Fixed effects for Motivation on spiroergometry and mechanography (mixed model analysis)

	Spiro maximal W/kg *p* value	Spiro RER peak *p* value	Spiro VO2 max *p* value	Peak Jump Force *p *value
Intercept	0.189	< 0.001	0.025	0.008
Treatment group	0.972	0.048	0.896	0.316
Time	0.125	0.478	0.145	0.326
Gender	0.003	0.395	< 0.001	< 0.001
Age	0.355	0.065	0.832	0.381
Treatment group * time	< 0.001	0.330	0.052	0.643
Motivation	0.029	0.005	0.141	0.075

Treatment group * time = treatment group over time

**Table 7:** Fixed Effects of MSES on DIKJ and BDI-II raw scores over time (mixed model analysis)

Source	DIKJ raw score *p* value	BDI-II raw score *p* value
Intercept	0.018	0.586
Treatment group	0.774	0.297
Time	< 0.001	< 0.001
Treatment group * time	0.991	0.573
Gender	0.408	0.098
Age	0.607	0.294
MSES	0.142	0.025

Treatment group * time = treatment group over time

### Influence of Motivation on Enjoyment (MSES scores)

Calculated with univariate ANOVA, motivation groups did not show a significant influence on enjoyment scores in MSES (supplement, table A8). Though compared to the Motivation group, MSES sum score was on average − 3.86 lower in the Demotivation group and − 4.62 lower in the Neutral group.

### Influence of MSES sum score and subscales on DIKJ and BDI-II over time

MSES sum scores showed a statistically significant effect on BDI-II (*p* = 0 0.025) but not on DIKJ (*p* = 0.142) in the mixed model analysis (Table 7). With every point more in the MSES enjoyment scale, DIKJ falls 0.21 points and BDI-II decreases 0.50 (Table A9). High rating in “Perceived competence” was near significance for a decrease in BDI-II (*p* = 0.054) and significant for DIKJ (*p* = 0.026) whereas the subscales “specific movement experience” and “enjoyment” showed an influence on BDI-II scores (*p* = 019 and *p* = 0.030), but not on DIKJ (table A10).

### Influence of MSES on physiological factors

A statistically significant influence of MSES sum scores on the physiological parameters (spiroergometry and mechanography) could not be demonstrated (table A11).

### Adverse events

Neither Adverse Events (AE), nor Serious Adverse Events (SAE) in relation to the interventions were reported. One subject suffered a metacarpals fracture due to self-injury, but continued trainings. Another suffered a skin infection but also continued exercising. One subject was moved to the closed ward because of elevated suicidality for one night, not related to the trainings, and could also continue exercising.

## Discussion

In the clinical study “Mood Vibes “, two vigorous exercise interventions (whole-body vibration and ergometer cycling) were applied in comparison to TAU during stationary treatment of adolescent depression. Both intervention groups responded more in the first 6 weeks to treatment measured by DIKJ scores, though the difference became significant only after week 26 [38]. In the present article, we investigated if motivational placebo-related factors could have played a substantial role in the treatment effects of the exercise conditions. Placebo-like effects are known to influence the psychological outcomes of exercise trainings [14]. Lindheimer et al. even suggested that the effect of exercise on psychological outcomes would be considerably less after correcting for the placebo responses [14].

Psychological factors recognized to be part of placebo responses are, e.g., beliefs, expectations, conditioning, learning, memory, reward and motivation, between others [30]. Two main mechanisms are especially supported in research literature. One is beliefs/ expectation regarding positive effects of a treatment. The other is positive conditioning, based on prior experiences [27, 30, 64]. There are some authors, who define a further aspect as a subset of expectations: the more implicit, subconscious expectancies [24]. Additionally some works focus on the interplay of expectations and positive conditioning [30]. Placebo-adherent effects are investigated in a broad range of medical conditions [27, 30, 65]. Beside the psychological mechanisms, the neurobiological mechanisms are in the mire. The influence of positive outcome expectations and conditioning on the release of endogenous opioids, hormones, immune cells or the changes of metabolic processes in different brain areas, are only a few to mention [30]. The peculiarity of placebo effects in children, which is also interesting, will not be discussed here, as our study population included only 13–18 years [15].

The main objective of the study “Mood Vibes” was to investigate the clinical effectiveness of an add-on exercise treatment in clinical inpatients regarding depression scores. Nevertheless, as secondary outcome parameters, we wanted to know if prior sports experiences would play a role in the sense of positive conditioning and/ or prevention. In addition, we wanted to know the influences of motivational factors towards exercising, including the beliefs and expectations with regard to exercise effects. In sum, we examined psychological factors, which are known to contribute to the placebo response, without a principal aim to focus on it.

As shown in the results, the prior sports experiences did not have the hypothesized influences on the depression scores in our sample. Not at baseline, nor in the longitudinal course of the study. We did not find a difference in the depression scores between the very active group, the medium active group and the less active group. We would have expected that prior positive experiences in sporting would have had a positive conditioning effect when engaging in a vigorous exercise program in the treatment of depression. As we did not apply experimentally induced expectations in our study, we expected that previous experiences from exercising would work as “habitual expectation” in the sense of a positive mindset [36]. The literature concerning the topic of habitual expectations is still contradictory [36, 66, 67]. Our findings rather point to no effect in this sense. Mothe et al. analyzed to which extent negative habitual expectations could be changed by induced positive expectations,. Although this point could have been interesting in our sample, it was not addressed. In Mothe’s study, expectation-manipulation showed effects only on the neurophysiological level, and as such could not confirm the positive results of Helfer et al. in this matter [37]. Concerning the “induced expectations”, we have to add that when informing about the study and also during the interventions, study staff tried to convey the expected positive effect of the applied physical exercises. Although we did not assess the influence of this mindset and communication attitude later on in our questionnaires. Moreover, one could argue that only due to participation in the intervention groups, a “Hawthorn effect” could have played a role in the outcomes of the exercise groups.

Numerous studies have analyzed, if regular exercising serves as a protective factor against depression [68–71]. Harvey et al. discussed that in their prospective cohort study, the results could suggest that up to 12% of depressive episodes could be prevented by exercising a minimum of one hour each week, regardless of the intensity of the exercise. The findings of our sample cannot support those results, as there was no significant difference between depression scores at baseline of those adolescents who exercised regularly and those who were inactive. Therefore, exercise in our clinical patient group seems to not have had a protective effect. On the other hand it has to be taken into account, that our sample of inpatients showed at baseline depression scores borderline between moderate and severe depression (see Supplements table A2). Therefore, it may be assumed that they have more treatment resistant depressive conditions and that exercise might be more effective at preventing mild to moderate depression.

Positive outcome expectancies of exercising, as well as further psychological motivators to exercise (e.g., “to be less sad”, “to be healthier”, “to be fitter”, “to be better looking”, “to deal better with aggressions”, “to distract from problems”, “to feel better in my body”, “to have social contacts” etc.), were assessed by the questionnaire “Motivations and Barriers to Sports” (MBS). The questionnaire did not target the expectationsconcerning the applied physical activities in the intervention groups, but the expectancies on physical activities in general. So we examined only the “habitual expectancies” [34] and motivations concerning sports and not the special expectations concerning the interventions. This is certainly a limitation to consider. Moreover, we did not assess if participating in an interventional exercise study with the objective to ameliorate depressive mood, has formed a study-specific expectation [72]. In our study we did not perform specific manipulations to generate experimentally-induced expectations [34], but unintended study-specific expectations generated through the study-exercise-participation, could be interpreted as incidentally-induced expectations, as specified by Lindheimer et al., 2019 [73]. This is certainly another limitation to bear in mind. The MBS covers not only expectations concerning corporal or mood states, but also social aspects and enjoyment factors. Depending of the ratings, we clustered the adolescents in three motivation groups: demotivated group, neutral group, motivated group. As we showed in the results, the motivation group, and the neutral group, improved significantly more regarding depression severity compared to the demotivation group. Our results suggest that it seems to be sufficient to have a neutral attitude towards exercising to benefit more. An explicit motivation to perform PA was not required to have a good clinical outcome. Presumably, an open-mindedness “opens the door” for a possible positive effect. This seems especially encouraging for the treatment of depressed adolescents with add-on vigorous exercise treatment, because in depressed mood it is especially difficult to achieve a positive motivation in this clientele. On the other hand, the knowledge that demotivated patients improve less from an add-on PA treatment has to also be taken into account. The implication is that the indication to apply an add-on exercise-treatment has to be differential.

In summary, our findings about the possible effects of motivational factors and expectations in exercise treatment of depression are in line with the majority of the existing literature, where expectations about treatment outcomes are seen as a part of placebo responses [74]. Interestingly, for significantly better results in the physiological outcomes of spiroergometry and mechanography (jump peak force), it was necessary to be in the motivation group, neutral attitude was not sufficient [14, 30, 64, 75].

Furthermore, the results show that higher scores in the Enjoyment Scales (MSES), influenced significantly the decrease in depressive scores measured by BDI-II. The strongest influence of the MSES subscales on BDI decrease was not in the subscale “enjoyment”, but in the scale “Specific Movement Experience”. The influence on DIKJ scores was only significant in the scale “perceived competence”. So the feeling of self-efficacy could even be more important than the feeling of joy in the execution of PA with an anti-depressant purpose [57, 76]. It was surprising, that the motivation group did not achieve significantly higher results in the Enjoyment Scales, as enjoyment is generally seen as dependent of positive expectations [53, 56, 57].

In conclusion, our results suggest, that positive expectations and beliefs towards exercising as well as enjoyment, are important motivational factors that influence the outcome of vigorous PA interventions in the treatment of depressed adolescents. As demonstrated, our study hints towards the importance of achieving at least a neutral attitude towards an exercise intervention as treatment option against depression. On the other hand, we could deduce that demotivated patients should not be treated with an exercise intervention, as they do not profit from this “alternative medication”. However, even if exercise for some patients will not help improvement in depression scores, it will have a panoply of profitable effects for the somatic and social conditions as well as for self-esteem matters [77, 78]. As motivation seems to be a clue factor, the challenge could be to develop strategies to modify beliefs, expectations and motivations about exercise, or to detect individually enjoyable exercise to increase motivation. More profound knowledge could contribute to use the psychological factors for maximizing treatment outcomes for the patients [30, 64, 65].

### Limitations

The primary objective (and so the calculated power) of the parent study was the clinical outcome of an add-on sports therapy for depressed adolescents. This must be considered as important limitation for the results on motivational confounders. Our findings should, therefore, be interpreted cautiously. Moreover, the sample size is small, even if this is due to the design of the parent study as a feasibility study. The limited available number of depressed adolescents in inpatient treatment could be resolved in the future by a multicenter design. The lack of randomization of the control group is another weakness which should be taken into account. The non-randomization of the control group did certainly contain a bias for the results of the study, even though the distribution of the activity-grades did not differ significantly between the groups. Furthermore, and perhaps most importantly, it would have been necessary to include an active sham control group. As we have seen, that the placebo inherent factors like expectation and positive beliefs can only be tested against an active sham-exercise group. Further studies in this area are of great interest and the recommendations of Beedie et al., for improved placebo research in physical activity treatments, should be taken into account [35].

## Supplementary Information

Below is the link to the electronic supplementary material.

Supplementary file1 (DOCX 240 KB)

## Data Availability

Data and material from the presented study can be facilitated by the corresponding author.
