# Supplementary material for: Influence of motivational placebo-related factors on the effects of exercise treatment in depressive adolescents
Source: Eur Child Adolesc Psychiatry. 2021 Mar 12;31(7):1–14. doi: 10.1007/s00787-021-01742-5 (PMC9343287; doi:10.1007/s00787-021-01742-5)
Supplement: Supplementary file 1 — Supplementary file1 (DOCX 240 KB) [file 787_2021_1742_MOESM1_ESM.docx]

***E-Supplements:***

*Table A1: Study design*

| **Screening**  Inpatient treatment  Major Depression (SKID I)  DIKJ> 18 raw points  No exclusion criteria  Meeting Inclusion criteria |  |  |
| --- | --- | --- |
| **3 Measure Points**  t0=Inclusion  t1= after 6 weeks intervention  t2= 8 weeks after t1 (no further intervention) | **Physical Measures**:  Spiroergometry including lactate blood levels  Jump mechanography  Calipermetry, BMI | **Psychological Parameters:**  Clinical interview (SKID I)  Depression questionnaires (DIKJ, BDI II)  Sport questionnaires (Motivation and Barriers to Sports, MSES)  Feed-Back questionnaire |
| **1 Post Measurement**  Post t2 =  3 months after t2 (no further intervention) |  | Depression questionnaires (DIKJ, BDI II) |

*See: Wunram et al., Eur Child Adolesc Psychiatry. 2018 May;27(5):645-662.*

*Table A2:* *Demographic and clinical characteristics at t0*

|  | Subjects | | | | | | | | | | | | | | | p | |
| --- | --- | --- | --- | --- | --- | --- | --- | --- | --- | --- | --- | --- | --- | --- | --- | --- | --- |
| **Data at t0** | Total  N=64 | | | Ergometer  n=20 | | | | WBV  n=21 | | | | Controls  n=23 | | | |  | |
| Sex ♂ | 18 | (28.1) |  | | 8 | (40) |  | | 6 | (28.6) |  | | 4 | (17.4) |  | | 0.268 |
| Age | 15.9 | ± 1.1 |  | | 16.1 | ± 1.2 |  | | 15.9 | ± 1.2 |  | | 15.7 | ± 1.1 |  | | 0.531 |
| BMI | 24.6 | ± 6.2 |  | | 26 | ± 7.6 |  | | 24.7 | ± 5.9 |  | | 23.3 | ± 5.0 |  | | 0.361 |
| IQ | 100.1 | ± 11.9 (4) |  | | 100.4 | ± 8.4 (1) |  | | 100.4 | ± 14.1 (1) |  | | 99.6 | ± 13.0 (2) |  | | 0.968 |
| DIKJ Score | 27.6 | ± 6.4 |  | | 27.0 | ± 6.2 |  | | 26.9 | ± 6.2 |  | | 28.8 | ± 6.9 |  | | 0.560 |
| BDI II Score | 29.6 | ± 11.9 |  | | 29.9 | ± 9.2 |  | | 26.8 | ± 13.1 |  | | 32.0 | ± 12.6 |  | | 0.466 |
| **Data at t1** |  |  |  | |  |  |  | |  |  |  | |  |  |  | |  |
| Length of stay | 68 | ± 33 |  | | 80 | ±36 |  | | 60 | ± 20 |  | | 64 | ± 39 |  | | 0.123 |
| Dropouts | 12 | (19) |  | | 3 | (4.8) |  | | 3 | (4.7) |  | | 6 | (9.3) |  | | 0.565 |
| Medication |  |  |  | |  |  |  | |  |  |  | |  |  |  | |  |
| None  PRN or SSRI < 3 weeks  > 3 weeks | 47  9  8 | (73.4)  (14.1)  (12.5) |  | | 14  5  1 | (70)  (25)  (5) |  | | 18  2  1 | (85.7)  (9.5)  (4.8) |  | | 15  2  6 | (65.2)  (8.7)  (26.1) |  | | 0.224  0.224  0.224 |
| Number of Trainings |  |  |  | | 23.5 | ± 2.47 (3) |  | | 22.1 | ± 3.7 (3) |  | |  |  |  | | 0.690 |
| Additional Sports/minutes | 476 | ± 567 |  | | 298 | ± 415 (13) |  | | 650 | ± 456 (15) |  | | 480 | ± 0 (22) |  | | 0.013 |
| Total therapy time/minutes | 1077 | ± 419 (13) |  | | 1149 | ± 419 (4) |  | | 979 | ± 381 (3) |  | | 1113 | ± 463 (6) |  | | 0.698 |
| Quantitative variables are summarized as mean ± SD (missing), p from one-way ANOVA; qualitative variables as n (%), p from Chi Square test or Fisher’s exact test | | | | | | | | | | | | | | | | | |
| PRN=Pro Re Nata | | | | | | | | | | | | | | | | | |
| *See: Wunram et al., Eur Child Adolesc Psychiatry. 2018 May;27(5):645-662.* | | | | | | | | | | | | | | | | | |

*Figure A1: Mean-DIKJ raw score for treatment groups over time (mixed model analysis)*


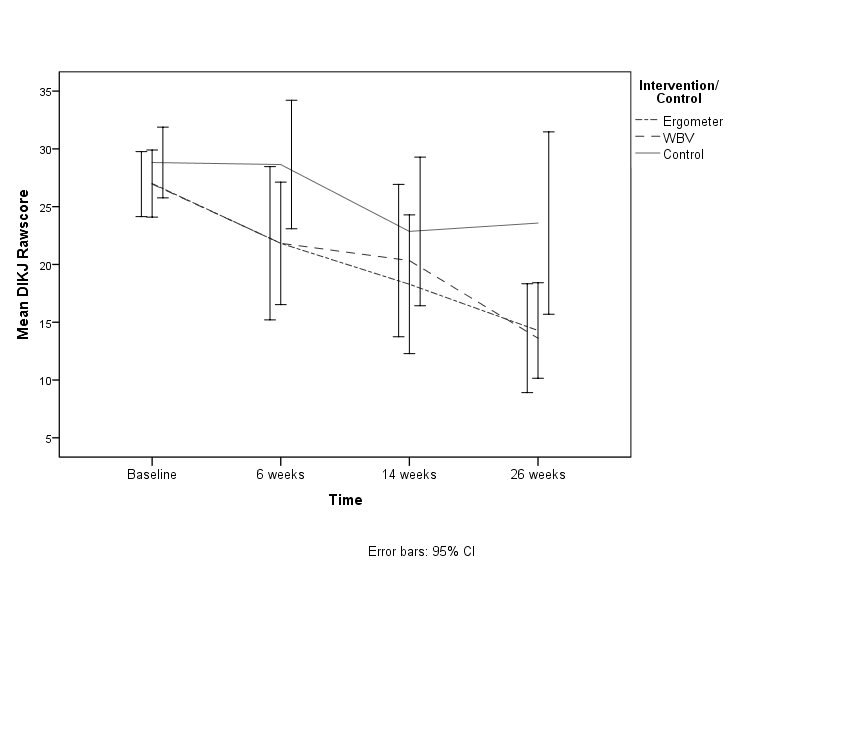


*See: Wunram et al., Eur Child Adolesc Psychiatry. 2018 May;27(5):645-662.*

*Table A3: Pairwise comparisons and effect Sizes DIKJ score intervention/ control*

| Pairwise Comparisons | | | | | | | |
| --- | --- | --- | --- | --- | --- | --- | --- |
| Time | Treatment group (I) | Treatment group (J) | Mean Difference (J-I) | SE | p-value | 95% Confidence Interval  for Difference | |
|  |  |  |  |  |  | Lower Bound | Upper Bound |
| Baseline | Control | Ergometer | -1.1 | 1.9 | 0.584 | -2.8 | 4.9 |
|  |  | WBV | -1.5 | 1.9 | 0.415 | -2.2 | 5.2 |
| 6 weeks (t1) | Control | Ergometer | -4.8 | 3.6 | 0.190 | -2.4 | 12.0 |
|  |  | WBV | -6.3 | 3.6 | 0.082 | -0.8 | 13.4 |
| 14 weeks (t2) | Control | Ergometer | -2.1 | 4.0 | 0.592 | -5.8 | 10.1 |
|  |  | WBV | -3.4 | 3.9 | 0.395 | -4.5 | 11.2 |
| 26 weeks  (post-t2) | Control | Ergometer | -8.1 | 3.8 | 0.037 | 0.5 | 15.8 |
|  |  | WBV | -8.0 | 3.8 | 0.042 | 0.3 | 15.7 |

*See also: Wunram et al., Eur Child Adolesc Psychiatry. 2018 May;27(5):645-662.*

*Table A4: Exercises of WBV-training and Ergometer training*

| **WBV: Exercise description** | frequency/ amplitude | Sessions  1-12 | Sessions  13 onward | **Ergometer training**  Intervals in minutes | % of maximal wattage in spiroergometry |
| --- | --- | --- | --- | --- | --- |
| “See-Saw”: Rocking the feet between the ball and [...] the heel | 20 Hz/ 2 | 2 min./2 min. pause | 3 min./ 3 min. pause | 3 min. | 40% |
| Squats: approximately 20 per minute | 20 Hz/ 2 | 2 min. ./2 min. pause | 3 min. ./ 3 min. pause | 6 min. | 50 % |
| „Tree in the wind“: Lateral side-movement with outstretched arms above the head | 20 Hz/ 2 | 2 min. ./2 min. pause | 3 min. ./ 3 min. pause | 3 min. | 70/80 % |
| „Rotation“: Lateral trunk-rotation of horizontally extended arms from left to right | 20 Hz/ 2 | 2 min. ./2 min. pause | 3 min. ./ 3 min. pause | 6 min. | 50 % |
| „Holding arms“: Arms 90 degree extended in front of the body and pulling a theraband | 20 Hz/ 2 | 2 min. ./2 min. pause | 3 min. ./ 3 min. pause | 3 min. | 70/80 % |
| „The Chair“: holding posture with 90 degrees bended knees like if sitting on a virtual chair | 20 Hz/ 2 | 2 min. ./2 min. pause | 3 min. ./ 3 min. pause | 6 min. | 50 % |
|  |  |  |  | 3 min. | 40 % |

*See also: Wunram et al., Eur Child Adolesc Psychiatry. 2018 May;27(5):645-662*

*Table A5: TAU-therapies (in minutes)*

| Length of stay in days  therapy time in minutes | Total [51] | Ergometer [16] | WBV [18] | Control [17] | p |
| --- | --- | --- | --- | --- | --- |
| Total therapy week 1-6 | 1077 (419) | 1149 (419) | 979 (381) | 1113 (463) | 0.46 |
| Total therapy week 7-14 | 410 (583) | 606 (784) | 268 (314) | 375 (566) | 0.23 |
| Psychotherapy week 1-6 | 311 (173) | 311 (157) | 309 (178) | 314 (193) | 0.99 |
| Psychotherapy week 7-14 | 108 (167) | 190 (243) | 71 (105) | 70 (104) | 0.06 |
| Art therapy week 1-6 | 304 (191) | 294 (161) | 314 (255) | 304 (143) | 0.95 |
| Art therapy week 7-14 | 122 (203) | 173 (230) | 89 (153) | 109 (224) | 0.47 |
| Sports therapy week 1-6 | 240 (266) | 256 (258) | 161 (171) | 308 (339) | 0.26 |
| Sports therapy week 7-14 | 97 (182) | 117 (227) | 42 (66) | 134 (212) | 0.29 |
| Group therapy week 1-6 | 32 (51) | 35 (41) | 26 (40) | 36 (71) | 0.80 |
| Group therapy week 7-14 | 16 (51) | 17 (49) | 5 (17) | 26 (73) | 0.50 |
| Music therapy week 1-6 | 36 (78) | 39 (113) | 26 (55) | 43 (58) | 0.81 |
| Music therapy week 7-14 | 10 (46) | 32 (127) | 9 (40) | 1 (4) | 0.47 |
| Ergo therapy week 1-6 | 11 (72) | 32 (127) | 0 | 4 (18) | 0.39 |
| Ergo therapy week 7-14 | 0 | 0 | 0 | 0 | - |
| Social Service week 1-6 | 55 (73) | 62 (78) | 54 (60) | 12 (23) | 0.90 |
| Social Service week 7-14 | 29 (69) | 41 (102) | 26 (55) | 43 (58) | 0.46 |
| Parents meetings week 1-6 | 86 (84) | 120 (83) | 89 (94) | 52 (61) | 0.70 |
| Parents meetings week 7-14 | 28 (54) | 48 (75) | 16 (42) | 22 (34) | 0.20 |
| Mean (SD); p from one-way ANOVA; [N] | | | | | |

*See also: Wunram et al., Eur Child Adolesc Psychiatry. 2018 May;27(5):645-662*

*Table A6: Descriptives Spiroergometry and Leonardo Mechanography*

| Group | | T0  maxWatt/ KG | T1 maxWatt/KG | T2 maxWatt/KG | T0 RERpeak | T1 RERpeak | T2 RERpeak | T0 Jump peak Watt/KG | T1 Jump peak Watt/KG | T2Jump peak/Watt/KG |
| --- | --- | --- | --- | --- | --- | --- | --- | --- | --- | --- |
| Ergometer | Mean | 1.81 | 2.17 | 2.13 | 24.59 | 25.78 | 29.95 | 38.73 | 38.39 | 39.80 |
|  | SD | 0.58 | 0.61 | 0.65 | 7.96 | 6.59 | 6.55 | 9.25 | 7.87 | 7.14 |
|  | Minimum | 0.70 | 1.08 | 1.26 | 12.55 | 14.66 | 21.18 | 18.10 | 22.43 | 30.98 |
|  | Maximum | 3.08 | 3.49 | 3.52 | 38.77 | 42.31 | 41.62 | 56.37 | 56.50 | 56.59 |
| WBV | Mean | 2.01 | 1.97 | 1.89 | 26.78 | 26.23 | 26.25 | 37.63 | 38.12 | 37.49 |
|  | SD | 0.56 | 0.63 | 0.55 | 7.76 | 6.92 | 8.69 | 6.87 | 6.59 | 6.68 |
|  | Minimum | 1.01 | 0.99 | 1.07 | 13.03 | 16.01 | 14.92 | 27.38 | 28.19 | 29.34 |
|  | Maximum | 3.08 | 3.44 | 3.36 | 39.76 | 43.03 | 44.20 | 52.69 | 48.36 | 49.46 |
| Control | Mean | 1.89 | 1.90 | 1.90 | 24.64 | 25.43 | 25.93 | 36.91 | 37.99 | 38.03 |
|  | SD | 0.52 | 0.53 | 0.51 | 8.27 | 7.15 | 5.93 | 8.17 | 9.55 | 10.36 |
|  | Minimum | 0.84 | 1.21 | 0.89 | 5.53 | 13.88 | 14.52 | 25.17 | 24.26 | 24.37 |
|  | Maximum | 3.09 | 3.25 | 3.18 | 42.03 | 42.13 | 41.15 | 58.87 | 60.85 | 59.73 |
| Total | Mean | 1.90 | 2.02 | 1.97 | 25.35 | 25.84 | 27.41 | 37.71 | 38.17 | 38.42 |
|  | SD | 0.55 | 0.60 | 0.58 | 7.95 | 6.75 | 7.32 | 8.04 | 7.87 | 7.88 |
|  | Minimum | 0.70 | 0.99 | 0.89 | 5.53 | 13.88 | 14.52 | 18.10 | 22.43 | 24.37 |
|  | Maximum | 3.09 | 3.49 | 3.52 | 42.03 | 43.03 | 44.20 | 58.87 | 60.85 | 59.73 |

*SD= Standard Deviation*

*See also: Wunram et al., Eur Child Adolesc Psychiatry. 2018 May;27(5):645-662*

*Table A7: Estimated marginal means for Motivation on spiroergometry and mechanography (mixed model analysis pairwise comparisons)*

|  | (I) Motivation | (J) Motivation | Mean Difference (I-J) | 95% Confidence Interval | | p-value |
| --- | --- | --- | --- | --- | --- | --- |
|  |  |  |  | Lower Bound | Upper Bound |  |
| Spiro  maxW/KG. | Motivation | Demotivation | 0.403 | 0.081 | 0.725 | 0.015 |
|  |  | Neutral | -0.352 | 0.026 | 0.678 | 0.035 |
| Spiro RER peak | Motivation | Demotivation | 0.092 | 0.029 | 0.156 | 0.005 |
|  |  | Neutral | 0.091 | 0.027 | 0.154 | 0.006 |
| Spiro  VO2peak | Motivation | Demotivation | 2.946 | -0.847 | 6.740 | 0.125 |
|  |  | Neutral | 3.472 | -0.309 | 7.253 | 0.071 |
| Peak Jump Force | Motivation | Demotivation | 3.662 | -0.191 | 7.515 | 0.062 |
|  |  | Neutral | 4.009 | 0.112 | 7.905 | 0.044 |

*Table A8: Influence of Motivation Group on MSES sum score in univariate ANOVA*

| **Parameter Estimates**  Dependent Variable: MSES sum score | | | | | |
| --- | --- | --- | --- | --- | --- |
| Parameter | B | SE | p-value. | 95% Confidence Interval | |
|  |  |  |  | Lower Bound | Upper Bound |
| Intercept | 22.482 | 1.863 | <0.001 | 18.678 | 26.286 |
| Demotivation | -3.857 | 2.886 | 0.191 | -9.750 | 2.036 |
| Neutral | -4.621 | 2.978 | 0.131 | -10.702 | 1.460 |
| Motivation | 0^a^ | . | . | . | . |
| a. This parameter is set to zero because it is redundant. | | | | | |

*Table A9: Estimates of MSES sum score on DIKJ and BDI in the mixed model analysis over time (other fixed effects not shown)*

| **Estimates of Fixed Effects^a^** | | | | | | |
| --- | --- | --- | --- | --- | --- | --- |
| Parameter | Estimates | SE | p-value | 95% Confidence Interval | |  |
|  |  |  |  | Lower Bound | Upper Bound |  |
| MSES on BDI II (dependent variable) | -0.495 | 0.212 | 0.025 | -0.924 | -0.067 |  |
| MSES on DIKJ (dependent variable) | -0.212 | 0.142 | 0.142 | -0.498 | 0.074 |  |

*Table A10: Fixed effects of MSES subscales on DIKJ and BDI (mixed model analysis over time, other fixed effects not shown)*

|  | **Type III Tests of Fixed Effects** | |
| --- | --- | --- |
| dependent variable | Source | p-value |
| BDI raw score | perceived competence | 0.054 |
|  | specific movement experience | 0.019 |
|  | enjoyment | 0.030 |
| DIKJ raw score | perceived competence | 0.026 |
|  | specific movement experience | 0.188 |
|  | enjoyment | 0.244 |

*A11 Fixed effects for MSES on spiroergometry and mechanography (mixed model analysis)*

|  | Spiro maximal W/kg  p-value | Spiro RER peak  p-value | Spiro VO2 max  p-value | Peak Jump Force  p-value |
| --- | --- | --- | --- | --- |
| intercept | 0.550 | 0.000 | 0.027 | 0.129 |
| treatment group | 0.927 | 0.101 | 0.728 | 0.868 |
| time | 0.064 | 0.502 | 0.157 | 0.893 |
| gender | 0.044 | 0.394 | 0.003 | 0.000 |
| age | 0.356 | 0.071 | 0.767 | 0.147 |
| treatment group*time | 0.001 | 0.266 | 0.064 | 0.447 |
| MSES | 0.635 | 0.341 | 0.798 | 0.968 |

*Table A12: Feedback questionnaire (FBQ)*

|  | Group | N | Mean | SD | SE | p |
| --- | --- | --- | --- | --- | --- | --- |
| Liked physical activity | Ergometer | 11 | 2.18 | 0.87 | 0.26 | 0.449 |
|  | WBV | 12 | 2.42 | 0.52 | 0.15 |  |
| Would have preferred the other intervention | Ergometer | 11 | 0.55 | 1.04 | 0.31 | 0.926 |
|  | WBV | 12 | 0.58 | 0.90 | 0.26 |  |
| Too many trainings (4 x/week) | Ergometer | 11 | 0.55 | 0.69 | 0.21 | 0.706 |
|  | WBV | 12 | 0.42 | 0.90 | 0.26 |  |
| Too long per unit (30 minutes) | Ergometer | 11 | 0.45 | 0.82 | 0.25 | 0.738 |
|  | WBV | 12 | 0.33 | 0.89 | 0.26 |  |
| Test battery too „hard“ | Ergometer | 11 | 1.09 | 0.94 | 0.29 | 0.201 |
|  | WBV | 12 | 0.58 | 0.90 | 0.26 |  |
| Motivation due to trainers | Ergometer | 11 | 2.55 | 0.52 | 0.16 | 0.706 |
|  | WBV | 12 | 2.42 | 0.99 | 0.29 |  |
| Would do the program as an outpatient | Ergometer | 11 | 1.27 | 1.19 | 0.36 | 0.895 |
|  | WBV | 12 | 1.33 | 0.99 | 0.28 |  |
| Would recommend it to others | Ergometer | 11 | 2.45 | 0.82 | 0.25 | 0.469 |
|  | WBV | 12 | 2.17 | 1.03 | 0.30 |  |
| Got motivated to continue exercise | Ergometer | 11 | 1.27 | 1.19 | 0.36 | 0.067 |
|  | WBV | 12 | 2.17 | 1.03 | 0.297 |  |
| Will come to the „post-exercise group“ | Ergometer | 11 | 0.73 | 1.19 | 0.36 | 0.844 |
|  | WBV | 11 | 0.64 | 0.92 | 0.30 |  |
| Got better mood due to program | Ergometer | 11 | 1.91 | 1.22 | 0.37 | 0.489 |
|  | WBV | 12 | 1.58 | 0.99 | 0.29 |  |
| Got better sensation toward own body | Ergometer | 10 | 2.30 | 0.95 | 0.30 | 0.134 |
|  | WBV | 12 | 1.58 | 1.17 | 0.34 |  |
| SD= Standarddeviation; SE: Standarderror of Means; p from t-test independent samples | | | | | | |
| *See also: Wunram et al., Eur Child Adolesc Psychiatry. 2018 May;27(5):645-662* | | | | | | |

*Figure A2: BDI II-raw score pairwise comparisons/ time*


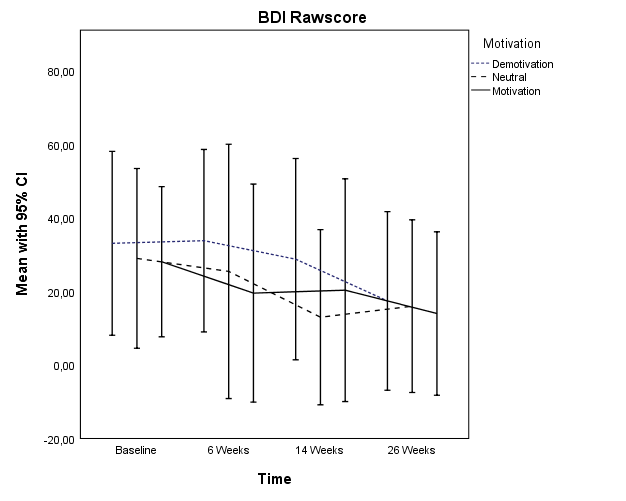


*Figure A3: Scatterplotts Activity against DIKJ scores at each measurement*

| 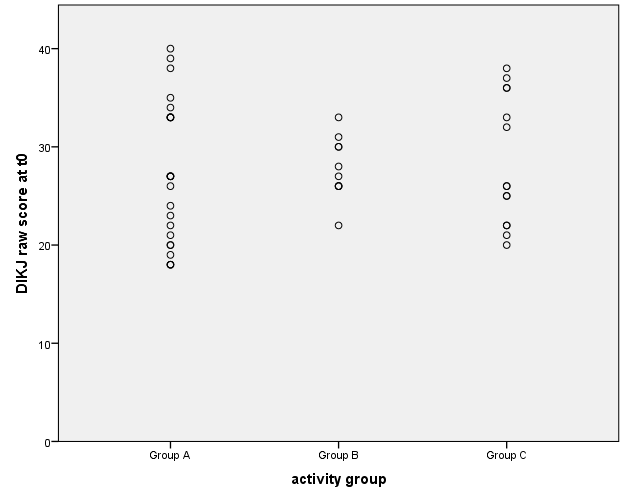 | 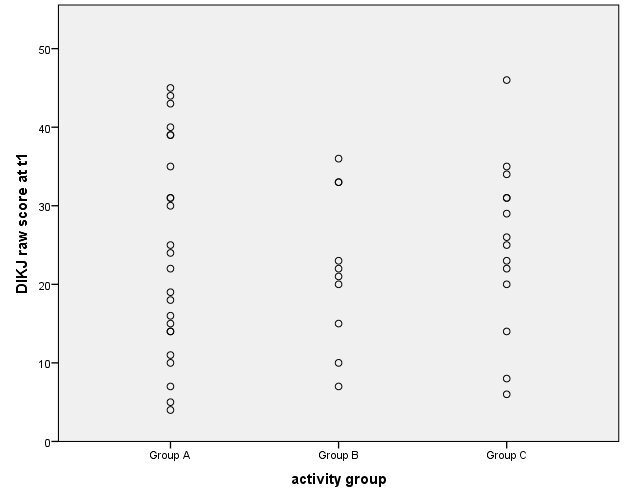 |
| --- | --- |
| 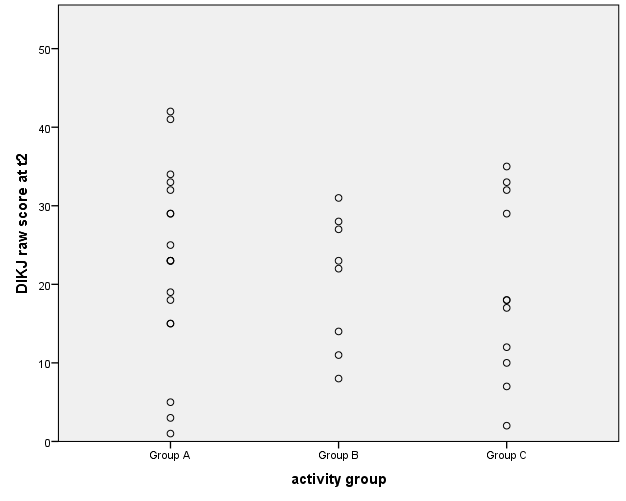 | 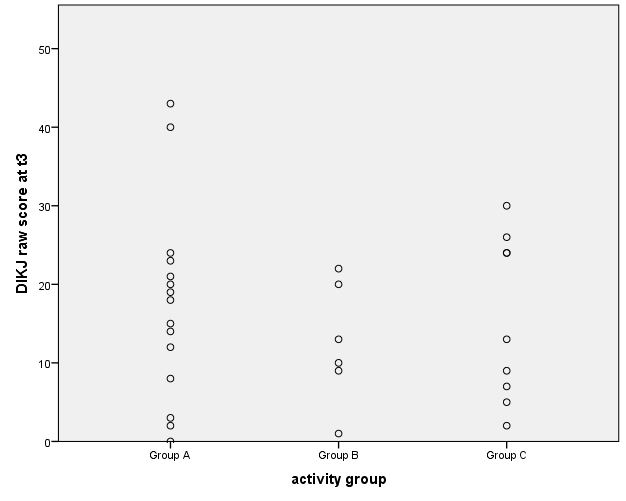 |

*Figure A4: Scatterplotts Motivation against DIKJ scores at each measurement*

| 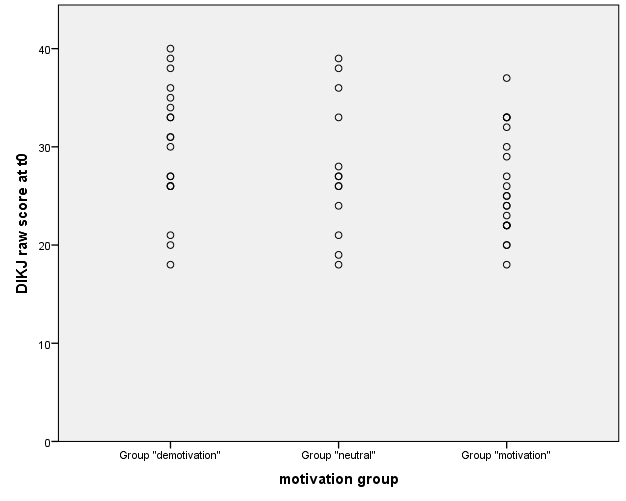 | 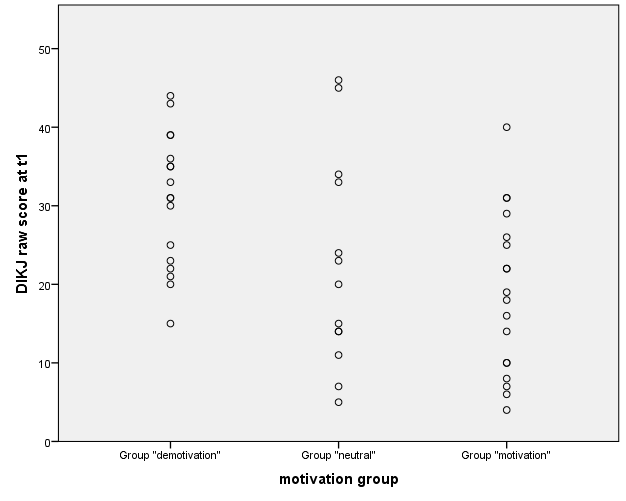 |
| --- | --- |
| 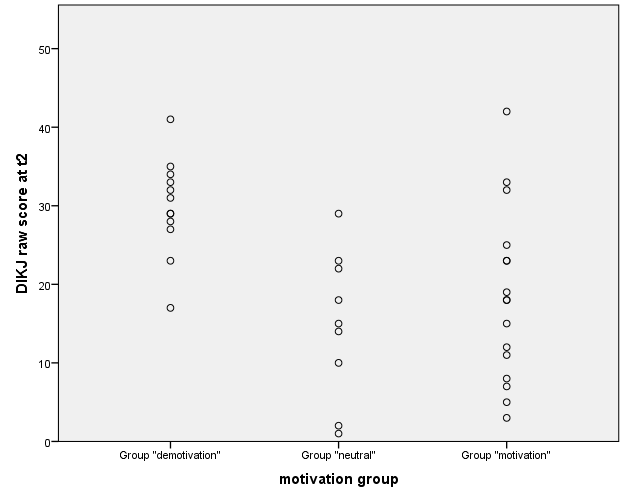 | 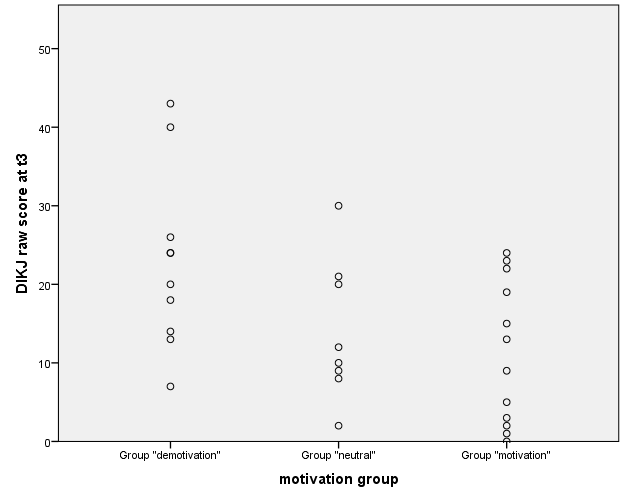 |

*Figure A5: Scatterplotts MSES against DIKJ scores at each measurement*

| 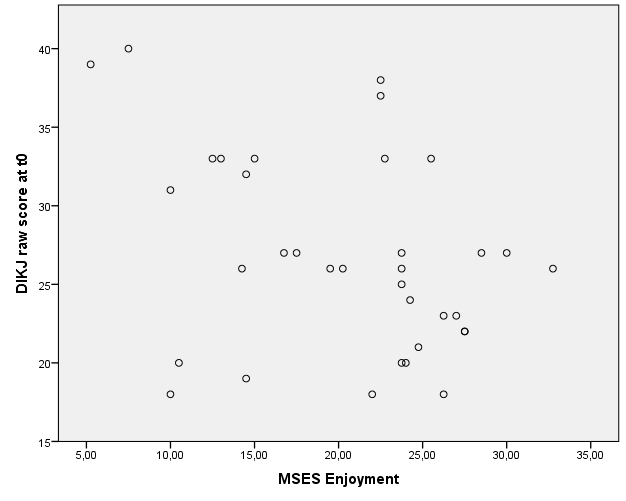 | 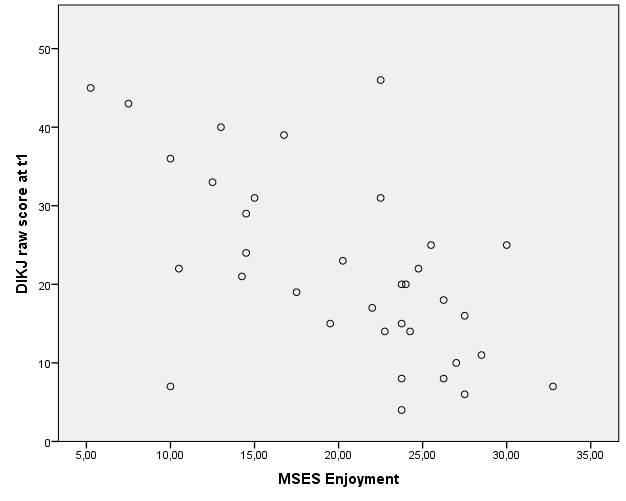 |
| --- | --- |
| 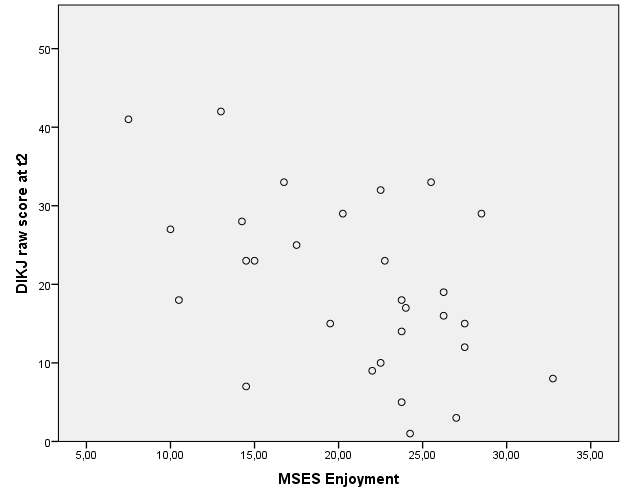 | 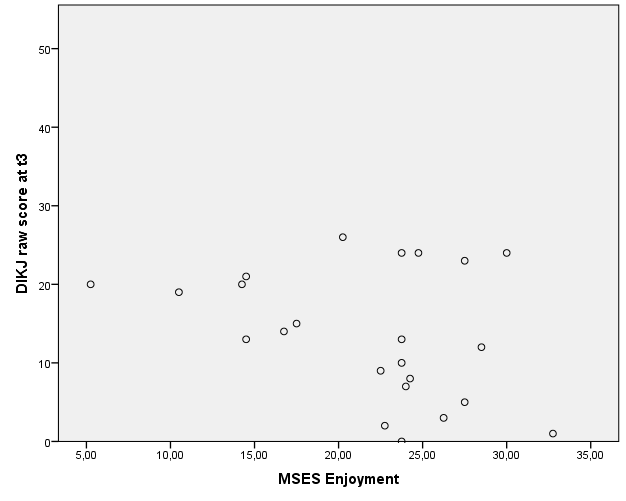 |
